# Differences in long-term mortality after out-of-hospital, in-hospital and intensive care unit cardiac arrests in finland

**DOI:** 10.1186/2197-425X-3-S1-A846

**Published:** 2015-10-01

**Authors:** I Efendijev, R Raj, M Reinikainen, S Hoppu, MB Skrifvars

**Affiliations:** Helsinki University Central Hospital, Helsinki, Finland; University of Helsinki, Helsinki, Finland; North Karelia Central Hospital, Joensuu, Finland; Tampere University Hospital, Tampere, Finland; University of Tampere, Tampere, Finland

## Introduction

Despite significant number of studies on cardiac arrest (CA), long-term outcome data are still scarce.

## Objectives

To determine long-term mortality after cardiac arrest in patients treated in Finnish university hospitals' intensive care units (ICU) stratified by arrest location.

## Methods

We performed a retrospective registry based study on adult patients treated in five Finnish university hospitals' ICUs between 2003 and 2013. We included patients with admission diagnosis of cardiac arrest according to the Acute Physiology and Chronic Health Evaluation III (APACHE III) and patients with positive value for Therapeutic Intervention Scoring System (TISS-76) item *“cardiac arrest and/or countershock within past 48 hours”*. Patients were classified by cardiac arrest location as out-of-hospital cardiac arrests (OHCA), in-hospital cardiac arrests (IHCA) and intensive care unit cardiac arrests (ICU-CA). Data on long-term outcome were retrieved from Finnish Population Register Centre. We excluded re-admissions and cases with missing baseline data. We calculated crude long-term mortality using life tables for the whole population and for patients who survived initial 30 days. The median time to death with interquartile range (IQR) was calculated for the non-survivors and compared between cardiac arrest location groups using Kruskal-Wallis test with pairwise comparison. In order to determine independent association of cardiac arrest location with mortality, we used Cox regression with case mix adjustment using pre-admission functional status, Simplified Acute Physiology Score II (SAPS II), chronic comorbidities, age, admission type (emergency and operative) and admission year.

## Results

Median follow-up time was 5.4 years (IQR 2.7-8.0). During the study period there were 2,413 OHCAs, 1,362 IHCAs and 1,952 ICU-CAs. For the general population one-year crude mortality was: OHCA 59%, IHCA 53% and ICU-CA 56%, five-year mortality was respectively: OHCA 67%, IHCA 64% and ICU-CA 67%. For patients with initial survival over 30 days one-year mortality was: OHCA 18%, IHCA 21%, ICU-CA 21% and five-year mortality was: OHCA 34%, IHCA 40%, ICU-CA 40%. Median time to death was 3.6 days (IQR 1.2-19.7) for OHCAs, 4.4 days (IQR 1.0-58.7) for IHCAs and 3 days (IQR 0.1-28.9) for ICU-CAs. There was significant difference for median time to death between ICU-CA and two other groups (*p* = 0.002 vs. OHCA and p < 0.001 vs. IHCA). In comparison to OHCA, IHCA was associated with a lower risk of death (hazard ratio [HR] 0.90, 95% confidence interval [CI] 0.82-0.99, *p* = 0.02), while risk of death for ICU-CA was similar (HR 1.09, 95% CI 1.00-1.19, *p* = 0.06).

## Conclusions

In this large retrospective study we have determined long-term mortality of cardiac arrest patients treated in university hospitals' ICUs stratified by arrest location. Among all ICU-treated CA patients, long-term survival of IHCA patients was better than that of OHCA and ICU-CA patients.

